# Cardiovascular comorbidities among public health clinic patients with diabetes: the Urban Diabetics Study

**DOI:** 10.1186/1471-2458-5-15

**Published:** 2005-02-08

**Authors:** Jessica M Robbins, David A Webb, Christopher N Sciamanna

**Affiliations:** 1 Philadelphia Department of Public Health, 500 South Broad Street, Philadelphia, PA, USA; 2 Jefferson Medical College, Philadelphia, PA, USA

## Abstract

**Background:**

We sought to determine the frequency and distribution of cardiovascular comorbidities in a large cohort of low-income patients with diabetes who had received primary care for diabetes at municipal health clinics.

**Methods:**

Outpatient data from the Philadelphia Health Care Centers was linked with hospital discharge data from all Pennsylvania hospitals and death certificates.

**Results:**

Among 10,095 primary care patients with diabetes, with a mean observation period of 4.6 years (2.8 after diabetes diagnosis), 2,693 (14.3%) were diagnosed with heart disease, including 270 (1.4%) with myocardial infarction and 912 (4.8%) with congestive heart failure. Cerebrovascular disease was diagnosed in 588 patients (3.1%). Over 77% of diabetic patients were diagnosed with hypertension. Incidence rates of new complications ranged from 0.6 per 100 person years for myocardial infarction to 26.5 per 100 person years for hypertension. Non-Hispanic whites had higher rates of myocardial infarction, and Hispanics and Asians had fewer comorbid conditions than African Americans and non-Hispanic whites.

**Conclusion:**

Cardiovascular comorbidities were common both before and after diabetes diagnosis in this low-income cohort, but not substantially different from mixed-income managed care populations, perhaps as a consequence of access to primary care and pharmacy services.

## Background

Patients with diabetes are at increased risk of a wide range of complications and comorbidities, which adversely affect quality of life, mortality, and health care services utilization. Cardiovascular diseases are the primary causes of morbidity and mortality among patients with diabetes, yet data on cardiovascular disease among patients with diabetes are limited [1]. Although microvascular pathologies, including retinopathy and nephropathy, have been shown to be strongly associated with glycemic control, macrovascular complications, including heart disease and cerebrovascular disease, appear to be less responsive to glycemic control, and strategies to reduce them are focussed on controlling other risk factors such as hypertension, hyperlipidemia, smoking, and obesity [2]. This complex array of risk factors and outcomes makes diabetes care exceptionally demanding both for patients and for health care providers.

Data on the prevalence and incidence of macrovascular comorbidities are largely derived either from clinical trials with selected patient populations, self-reported cross-sectional survey data, or managed-care studies of insured patients of higher socioeconomic status. The rising prevalence of diabetes in low-income inner-city populations underscores the need to understand the frequency with which these comorbidities occur in such populations and to assess possible disparities in their occurrence.

The Philadelphia Health Care Centers (HCCs) provide primary care and pharmacy services to approximately 100,000 patients annually through eight neighborhood centers and one sexually transmitted disease clinic. Individual patients are not billed for any services provided. Although the HCCs are available to all Philadelphia residents, their patients are almost universally low-income; over 60% have no health insurance. A majority of HCC patients are African American, but there are also substantial numbers of non-Hispanic white, Hispanic, and Asian patients. Diabetes is one of the most common diagnoses among adult HCC patients. Since to our knowledge no previous study has reported the prevalence or incidence of cardiovascular diseases in a comparable, ethnically diverse, low-income diabetic population, we sought to determine the frequency and distribution of cardiovascular comorbidities among HCC patients with diabetes.

## Methods

The Urban Diabetics Study cohort consists of 10,095 patients diagnosed with diabetes in the HCCs between January 1, 1996 and December 31, 2001. All diabetic patients were included except those for whom Social Security numbers were unavailable (less than 6% of all diabetic patients). All HCC visits between March 1, 1993 and December 31, 2001, all hospital discharges from any Pennsylvania hospital between January 1, 1993 and December 31, 2001, and death records have been linked for these patients. The data includes outpatient visits and hospitalizations both before and after the initial diabetes diagnosis, within the study time period. Race/ethnicity was classified based on the outpatient records. Diabetes incidence was calculated based on the admission date of the first hospitalization for which a diabetes diagnosis was recorded or the date of the first HCC outpatient visit for which a diabetes diagnosis was recorded, whichever came first.

The cardiovascular comorbidities assessed included any heart disease (ICD9 codes 402 and 410–429, ICD10 codes I5-I9, I11, I13, I20-I27, and I30-I52), myocardial infarction (MI) (ICD9 code 410, ICD10 codes I21-I23), congestive heart failure (CHF) (ICD9 code 428, ICD10 code I50), cerebrovascular disease (ICD9 codes 430–438, ICD10 code I6), and hypertension (ICD9 codes 401–405, ICD10 codes I10-I15). For each comorbid condition we assessed baseline prevalence at or before first diabetes diagnosis, incidence rate per 100 person-years of follow-up time following first diabetes diagnosis among those free of the condition at baseline, and cumulative prevalence, i.e., any occurrence over the course of the observation period. Logistic regression and proportional hazards regression were used to simultaneously assess multiple demographic risk factors, including sex, race/ethnicity, exact age, and year of diabetes incidence. Patients with unknown race/ethnicity (n = 7) were excluded from multivariable analyses.

## Results

Demographic characteristics of the 10,095 patients included are shown in Table 1. Mean age at diabetes diagnosis was 49.2 years, and mean observation period was 4.59 years, including 1.74 years before and 2.85 years after first known diabetes diagnosis. Among the patients for whom income was ascertained, median income was $7,092; 86% had annual incomes below $15,000. Table 2 shows which of the linked data sources provided diagnoses of each comorbidity.

**Table 1:** Demographic characteristics of Urban Diabetics Study cohort

	Total	African American	Hispanic	Non-Hispanic White	Asian	Other	Unknown
	**N**	**N**	**N**	**N**	**N**	**N**	**N**

Total	10095	7243	1119	1056	394	276	7
Females	5671	4230	528	540	224	145	4
Age							
0–4	17	12	2	2	1	0	0
5–14	38	28	2	2	4	2	0
15–24	213	149	25	28	8	3	0
25–34	477	359	52	41	11	14	0
35–44	1086	826	89	97	50	23	1
45–54	1614	1214	161	151	50	35	3
55–64	1542	1139	142	146	72	41	2
65–74	508	362	44	55	21	25	1
75–84	149	114	11	16	6	2	0
85+	30	27	0	2	1	0	0
Year of Diabetes Diagnosis							
1996	948	720	79	90	45	14	0
1997	969	714	78	116	40	20	1
1998	817	604	77	88	23	24	1
1999	854	637	92	74	26	25	0
2000	926	699	84	75	35	33	0
2001	1157	856	118	97	55	29	2
							
Males	4424	3013	591	516	170	131	3
Age							
0–4	28	26	0	1	1	0	0
5–14	78	68	7	2	1	0	0
15–24	419	371	20	22	3	3	0
25–34	1174	1015	81	55	15	7	1
35–44	2567	2243	155	113	29	26	1
45–54	3183	2797	163	132	52	38	1
55–64	2807	2467	119	134	45	42	0
65–74	902	800	36	32	20	14	0
75–84	290	254	9	23	3	1	0
85+	58	54	1	2	1	0	0
Year of Diabetes Diagnosis							
1996	732	509	92	101	17	13	0
1997	743	496	96	101	27	22	1
1998	692	486	95	74	21	16	0
1999	638	423	80	84	24	27	0
2000	694	457	103	75	33	25	1
2001	925	642	125	81	48	28	1

**Table 2:** Data sources for cardiovascular comorbid conditions

	**Outpatient only**	**Hospital only**	**Death Certificate only**	**Outpatient And Hospital**	**Outpatient and Death Certificate**	**Hospital and Death Certificate**	**Outpatient, Hospital, and Death Certificate**
Heart Disease	1125	700	38	731	9	31	59
Congestive Heart Failure	330	314	6	246	2	7	7
Myocardial Infarction	5	240	13	3	0	9	0
Cerebrovascular Disease	109	333	9	123	0	11	3
Hypertension	5248	218	0	2296	9	3	30

### Heart disease

Baseline prevalence of any heart disease was 14.8 %, including 4.1% with CHF and 1.0% with MI (Table 3). In multiple regression analyses, non-Hispanic white race/ethnicity (compared to the African-American reference group) was associated with substantially higher baseline prevalence of MI (Table 4). Hispanic race/ethnicity was associated with lower baseline prevalence of CHF, and Asian race/ethnicity was associated with lower baseline prevalences of MI, CHF, and any heart disease. Age and male sex were positively associated with baseline prevalence of MI, CHF, and any heart disease. Year of diabetes diagnosis was negatively associated with baseline prevalence of MI, CHF, and any heart disease, indicating that baseline prevalences declined between 1996 and 2001.

**Table 3:** Baseline prevalence, incidence, and cumulative prevalence of cardiovascular comorbid conditions

**Comorbidities**	**Total Cases**	**Cumulative Prevalence**	**Baseline Prevalence At or Before Initial Diabetes Diagnosis**		**Incident Cases After Diabetes Diagnosis**		**Incidence Rate**
	**N**	**%**	**N**	**%**	**N**	**% of those at risk**	**per 100 Person-Years**

Heart Disease	2,693	26.7%	1,495	14.8%	1,198	13.9%	5.36
Congestive Heart Failure	912	9.0%	417	4.1%	495	5.1%	1.85
Myocardial Infarction	270	2.7%	104	1.0%	166	1.7%	0.59
Cerebrovascular Disease	588	5.8%	294	2.9%	294	3.0%	1.07
Hypertension	7,804	77.3%	5,226	51.8%	2,578	52.9%	26.46

**Table 4:** Associations with baseline prevalence of comorbid conditions

	**Odds Ratio, (95% Confidence Interval)**						
	**Male**	**White**	**Hispanic**	**Asian**	**Other**	**Age (per 10 years)**	**Year of Diabetes Incidence**

Heart Disease	1.24 (1.10–1.39)	1.32 (1.11–1.57)	0.59 (0.48–0.73)	0.45 (0.31–0.65)	0.49 (0.32–0.74)	1.73 (1.66–1.81)	1.02 (0.99–1.05)
Congestive Heart Failure	1.33 (1.08–1.63)	0.86 (0.63–1.19)	0.28 (0.17–0.48)	0.37 (0.18–0.75)	0.32 (0.13–0.79)	1.76 (1.63–1.89)	1.06 (1.00–1.12)
Myocardial Infarction	1.36 (0.92–2.01)	1.94 (1.19–3.14)	0.52 (0.23–1.21)	0.00 (0.00->9.99)	0.31 (0.04–2.26)	1.47 (1.28–1.70)	1.03 (0.92–1.15)
Cerebrovascular Disease	1.46 (1.15–1.85)	0.90 (0.61–1.32)	0.64 (0.41–1.00)	0.56 (0.27–1.14)	0.10 (0.01–0.70)	1.89 (1.73–2.06)	1.01 (0.94–1.08)
Hypertension	0.75 (0.69–0.82)	0.69 (0.60–0.79)	0.39 (0.34–0.45)	0.32 (0.26–0.41)	0.30 (0.23–0.40)	1.85 (1.78–1.91)	1.85 (1.78–1.91)

Odds ratios are adjusted for other variables shown in the table: sex, race/ethnicity, exact year of age, and year of diabetes incidence. Reference groups for odds ratios are: Females (for Males); African Americans (for White, Hispanic, Asian, and Other); lower age; and earlier year of diabetes diagnosis.

Incidence of heart disease, among those who had not been diagnosed with it at the time of their diabetes diagnosis, was 5.36 cases per 100 person-years, with 13.9% of those at risk being diagnosed before the end of the study period (Table 3). Incidence of MI was 0.59 per 100 person-years. Incidence of CHF was 1.85 per 100 person-years. In multiple regression analyses (Table 5), Non-Hispanic whites, as compared to African-Americans, were at higher risk of MI and all heart disease. Hispanic and Asian patients were at lower risk of CHF. Incidence of heart disease increased slightly with later year of diabetes diagnosis. Age at diabetes diagnosis was associated with higher incidence of all heart disease outcomes, as was male sex.

**Table 5:** Risk factors for incident comorbid conditions

	**Hazard Ratio, (95% Confidence Interval)**						
	**Male**	**White**	**Hispanic**	**Asian**	**Other**	**Age (per 10 years)**	**Year of Diabetes Incidence**

Heart Disease	1.34 (1.19–1.50)	1.26 (1.06–1.50)	1.00 (0.84–1.20)	0.83 (0.62–1.13)	0.63 (0.42–0.95)	1.48 (1.42–1.54)	1.07 (1.03–1.12)
Congestive Heart Failure	1.57 (1.31–1.87)	1.10 (0.85–1.43)	0.57 (0.40–0.81)	0.55 (0.31–0.98)	0.99 (0.59–1.67)	1.56 (1.46–1.67)	1.06 (0.99–1.13)
Myocardial Infarction	1.71 (1.25–2.32)	1.81 (1.23–2.67)	0.95 (0.56–1.61)	0.47 (0.15–1.49)	0.45 (0.11–1.84)	1.56 (1.40–1.75)	1.01 (0.90–1.15)
Cerebrovascular Disease	1.20 (0.96–1.52)	1.08 (0.77–1.51)	0.61 (0.39–0.97)	0.47 (0.21–1.05)	0.56 (0.23–1.35)	1.66 (1.53–1.82)	0.99 (0.90–1.09)
Hypertension	1.04 (0.96–1.13)	0.71 (0.62–0.81)	0.67 (0.59–0.75)	0.78 (0.65–0.94)	0.61 (0.49–0.76)	1.32 (1.28–1.36)	1.29 (1.25–1.33)

Hazard ratios are adjusted for other variables shown in the table: sex, race/ethnicity, exact year of age, and year of diabetes incidence. Reference groups for hazard ratios are: Females (for Males); African Americans (for White, Hispanic, Asian, and Other); lower age; and earlier year of diabetes diagnosis.

Through the entire study period, 2,693 patients (26.7%) were diagnosed with heart disease (Table 3). MI was diagnosed in 270 patients (2.7%) and CHF in 912 (9.0%). In multiple regression analyses (Table 6), Non-Hispanic white race/ethnicity was associated with higher cumulative prevalence of MI (odds ratio [OR] 1.90) and any heart disease (OR 1.32). Hispanic race/ethnicity was associated with lower cumulative prevalence of CHF and any heart disease (OR 0.78, 95% CI 0.66–0.91). Asian race/ethnicity was associated with lower cumulative prevalences of all heart disease outcomes. Age at baseline and male sex were positively associated with cumulative prevalence of MI, CHF, and any heart disease. Year of diabetes diagnosis was negatively associated with cumulative prevalence of each of the heart disease outcomes.

**Table 6:** Associations with cumulative prevalence of comorbid conditions

	**Associations with Cumulative Prevalence of Comorbidities Odds Ratio, (95% Confidence Interval)**						
	**Male**	**White**	**Hispanic**	**Asian**	**Other**	**Age (per 10 years)**	**Year of Diabetes Incidence**

Heart Disease	1.33 (1.21–1.46)	1.32 (1.14–1.53)	0.78 (0.66–0.91)	0.58 (0.45–0.76)	0.52 (0.38–0.72)	1.71 (1.65–1.78)	0.85 (0.82–0.87)
Congestive Heart Failure	1.48 (1.28–1.71)	0.99 (0.79–1.23)	0.43 (0.31–0.58)	0.44 (0.28–0.70)	0.66 (0.41–1.05)	1.68 (1.59–1.78)	0.85 (0.82–0.88)
Myocardial Infarction	1.56 (1.22–2.00)	1.90 (1.39–2.59)	0.78 (0.50–1.23)	0.27 (0.09–0.85)	0.40 (0.13–1.26)	1.53 (1.39–1.67)	0.81 (0.75–0.87)
Cerebrovascular Disease	1.32 (1.11–1.57)	0.99 (0.76–1.29)	0.62 (0.44–0.86)	0.49 (0.28–0.86)	0.31 (0.13–0.70)	1.80 (1.69–1.93)	0.82 (0.78–0.86)
Hypertension	0.87 (0.79–0.97)	0.58 (0.49–0.68)	0.41 (0.36–0.48)	0.39 (0.30–0.49)	0.33 (0.25–0.43)	1.85 (1.78–1.93)	0.91 (0.88–0.93)

Odds ratios are adjusted for other variables shown in the table: sex, race/ethnicity, exact year of age, and year of diabetes incidence. Reference groups for hazard ratios are: Females (for Males); African Americans (for White, Hispanic, Asian, and Other); lower age; and earlier year of diabetes diagnosis.

### Cerebrovascular disease

Cerebrovascular disease was diagnosed in 588 patients, evenly split between cases diagnosed at baseline and incident cases subsequent to diabetes incidence (Table 3). Baseline prevalence of cerebrovascular disease was 2.9%. In multiple regression analyses (Table 4), Hispanic, Asian, and "other" race/ethnicities were associated with lower baseline prevalences of cerebrovascular disease. Age at baseline and male sex were positively associated with baseline prevalence of cerebrovascular disease, while year of diabetes diagnosis was negatively associated with baseline prevalence of cerebrovascular disease (OR 0.78, 95% CI 0.74–0.83).

The incidence rate for cerebrovascular disease, among those free of it at baseline diabetes diagnosis, was 1.07 per 100 person-years. In multiple regression analyses, only Hispanic race/ethnicity was associated with lower risk. Age was the only variable associated with a higher incidence of cerebrovascular disease.

Cumulative prevalence of cerebrovascular disease was 5.8%. Hispanic and Asian race/ethnicities were associated with lower cumulative prevalences of cerebrovascular disease, as was year of diabetes diagnosis. Age at baseline and male sex were positively associated with cumulative prevalence of cerebrovascular disease.

### Hypertension

A majority of patients (51.8%) had a diagnosis of hypertension at baseline (Table 3). Non-Hispanic white, Hispanic, Asian, and "other" race/ethnicities were all associated with lower baseline prevalence of hypertension, as was male sex (Table 4). Age and year of diabetes diagnosis were associated with higher baseline prevalence.

An additional 2,578 patients were diagnosed with hypertension after diabetes diagnosis, for a cumulative prevalence of 7,804 (77.3%). The incidence rate was 26.46 cases per 100 person-years. Non-Hispanic white, Hispanic, Asian, and "other" race/ethnicities were all associated with lower incidence of hypertension than the African-American reference group, while age and later date of diabetes diagnosis were associated with higher incidence.

Cumulative prevalence of hypertension was negatively associated with non-Hispanic white, Hispanic, Asian, and "other" race/ethnicities, male sex and year of diabetes diagnosis, and positively associated with age.

## Discussion

Patients in the Urban Diabetics Study cohort, like other diabetic patient populations, faced a substantial burden of cardiovascular comorbidity. Many were affected by comorbid conditions before they were diagnosed with diabetes, including 14.8% with preexisting heart disease, 2.9% with preexisting cerebrovascular disease, and 51.8% with preexisting hypertension. Among those free of these comorbidities at the time of diabetes diagnosis, similar proportions went on to develop them during followup.

The diabetic patients served by the Philadelphia HCCs are almost uniformly low-income and uninsured or underinsured. A large majority are African American. Nonetheless, the prevalence and incidence of major cardiovascular complications does not, in general, appear to exceed the rates of these complications in other patient populations, whether assessed in nationally-representative surveys or in insured, predominantly middle class managed care populations. Disparities associated with race/ethnicity were in varying directions. Non-Hispanic whites had lower rates of hypertension than African Americans but higher rates of heart disease, especially MI, while Asians and Hispanics had more favorable outcomes on several measures.

As expected, age was positively associated with all cardiovascular comorbidities. Male sex was associated with higher incidence and prevalence of all comorbidities except hypertension.

These findings are comparable to those for other diabetic populations not limited to low-income or uninsured patients. The cumulative prevalence of any heart disease in this study population (26.7%) was similar to the 24.5% prevalence of self-reported coronary heart disease among diabetic patients in the nationally representative 1999–2001 National Health Interview Surveys (NHIS), while the cumulative prevalence of cerebrovascular disease in our study (5.8%) was substantially lower than self-reported stroke in the NHIS sample (9.3%) [3]. However, the age distribution of diabetics in the NHIS sample was substantially older than in this population. If the comparison is restricted to NHIS diabetic patients age 35–64, national prevalences were 31% lower than in our population for heart disease (18.4% vs. 26.7%) and almost identical for cerebrovascular disease (5.9% vs. 5.8%). The overall incidence rate of MI (0.59 per 100 person-years) in the Urban Diabetics cohort was lower than that in the Kaiser Permanente managed care population studied by Karter, et al. [4]. Again, this may reflect the fact that the Urban Diabetics cohort was younger (mean age 49.2 years vs. 59.8 years). When the overall incidence rate in our population is multiplied by the hazard ratio for 10 years additional age in our study, the result (0.92 per 100 person-years) is almost identical to the age- and sex-adjusted rate for African Americans reported by Karter, et al. (0.91 per 100 person-years). The incidence rate of CHF, on the other hand, was higher in our population than in studies of Kaiser Permanente patients [5], while that of cerebrovascular disease was similar to the incidence of stroke reported by Karter, et al. Another study conducted with the Kaiser Permanente diabetes patients found evidence of hypertension for 74% of those diagnosed with diabetes [6], similar to the 77% cumulative prevalence in the Urban Diabetics study.

Other studies have reported somewhat divergent findings for heart disease [7,8] and cerebrovascular disease [9-11], but these studies involved either younger type 1 diabetic patients [7], earlier time periods [8], an older population [9], or analyses that excluded some patients with prior histories of cardiovascular disease [10,11]. Several studies have reported high rates of hypertension among diabetic patients, although none as high as the cumulative prevalence in this cohort [9,11-14].

Many of our findings on racial/ethnic differences in the incidence of MI, CHF, and cerebrovascular disease among diabetic patients are also similar to those found by Karter, et al. Like them, we found higher risks of MI for non-Hispanic whites than for other groups, and lower risks of CHF and cerebrovascular disease for Hispanics and Asians than for African Americans and non-Hispanic whites. The magnitudes of the differences between African-American and non-Hispanic white rates were broadly similar between the two studies, while rates for Asians and Hispanics were more variable. The 1999–2001 NHIS also found the prevalence of coronary and other heart disease among patients with diabetes (based on self-reported data) highest among non-Hispanic whites and lowest among Hispanics (rates for Asians were not reported) [3]. Unlike our study, the NHIS analyses found rates of cerebrovascular disease higher among African Americans with diabetes than among either Hispanics or non-Hispanic whites. Among diabetic patients in the Veterans Health Administration, rates of cardiovascular disease were lower for African Americans, Hispanics, and Asians than for non-Hispanic whites [9]. However, that study was restricted to patients with at least 4 outpatient clinic visits in a 12-month period, and race/ethnicity was not ascertained for 21.4% of the patients in that study, leaving considerable potential for bias in these results.

In our study, male sex was associated with higher baseline prevalence of all comorbidities, higher incidence of the heart disease comorbidities, and higher cumulative prevalence of all comorbidities except hypertension. Our findings are broadly consistent with those in other diabetic populations [3,4,15].

The similarities of our findings to those from the Kaiser Permanente studies are striking, given that the latter assessed an insured, West Coast cohort in which most Hispanics were Mexican American [4], while our study population was an overwhelmingly poor, uninsured or underinsured, East Coast cohort in which most Hispanics were Puerto Rican. The national origins of the Asian patients in the two studies probably also differ, as the Philadelphia Asian community includes larger proportions of South and Southeast Asians and smaller proportions of Japanese and Filipino individuals than the Asian communities of northern California [16]. Among both Hispanics and Asian Americans, national groups vary widely in socioeconomic status and health outcomes [17-19].

Our study is based on administrative records from several sources, each of which may contain errors, and is unlikely to capture as many cardiovascular endpoints than studies with study-specific, patient-level data collection [20,21]. Because the outpatient encounter forms allowed a maximum of four diagnostic codes, there may have been underreporting of some comorbidities, although cardiovascular disease was unlikely to go unrecorded. Outpatient care outside of the eight Philadelphia HCCs and hospitalizations outside of Pennsylvania were not ascertained. The design of the study could therefore undercount comorbid conditions and cardiovascular endpoints for patients who acquired health insurance or moved out of the city and therefore changed outpatient providers. As a test of the sensitivity of the results to migration in and out of the health system, we repeated the analyses, restricting the follow-up time from the dates of the first to the last to outpatient visit; the results were not materially changed.

Among the strengths of the study are its inclusion of a wide range of complications, 8+ year longitudinal design, large sample size, and the combination of data sources to enhance endpoint ascertainment. Our design captured diabetes diagnoses and cardiovascular comorbidity and endpoint data from three complementary sources: outpatient visits in any of the eight Philadelphia HCCs, inpatient visits within any hospital in Pennsylvania and death certificate records. Table 2 supports the observation of Kashner and colleagues that the addition of outpatient administrative data resulted in significantly higher estimates of comorbidity prevalence, strongly suggesting that it has value as an additional data source [22].

By limiting the study to patients with an initial diabetes diagnosis after the first 34 months for which we have data, we were able to ascertain the prevalence of cardiovascular conditions before and at the time of diabetes diagnosis as well as incident conditions diagnosed after diabetes incidence. We are unaware of any study that has presented similar data except for one limited to elderly African Americans and whites in North Carolina, which included only 653 patients with diabetes [23]. The fact that patients included in the study had no record of a diabetes diagnosis either in the Philadelphia Health Care Centers or in a hospital discharge record between March 1993 and the date of their diabetes diagnosis, which was no earlier than January 1996, should ensure that the great majority of patients included here were incident diabetes cases.

There is a strong, well-established association between socioeconomic disadvantage and cardiovascular disease in industrialized populations [24], including diabetic populations [25]. The absence of excess cardiovascular disease beyond that experienced in other diabetic patient populations in this disadvantaged cohort suggests that some factor has offset this disadvantage for this cohort. One possibility is that the provision of primary care, other outpatient services to which patients are referred by their primary care physicians, and prescription medications without out-of-pocket costs to all patients in the Philadelphia HCCs removes a significant barrier to effective care. Restricted use of medications due to costs is common [26] and has been shown to lead to poorer outcomes for several chronic diseases, including cardiovascular diseases [27,28].

This is the only study of which we are aware to report on cardiovascular comorbidity in a large, ethnically diverse cohort of low-income patients with diabetes with near-universal ascertainment of race/ethnicity. It includes substantial populations of low-income Asians, Hispanics, and non-Hispanic whites as well as African Americans, and allows comparisons of the experiences of these groups. The data is longitudinal and includes both outpatient and hospital discharges over an extended period. By looking at comorbid conditions both before and after diabetes incidence, we have provided both a picture of the health status of these patients at the initial presentation of diabetes and estimates of the incidence of additional comorbidities while being treated for diabetes.

## Conclusions

Cardiovascular comorbidities were common both before and after diabetes diagnosis in this low-income cohort, but not substantially different from mixed-income managed care populations. It is possible that access to primary care and pharmacy services without fees to individuals in this public health clinic system prevented the poorer outcomes usually seen for disadvantaged patients.

## Competing interests

The author(s) declare that they have no competing interests.

## Authors' contributions

JMR and DAW conceived the study and obtained the data. JMR designed the study, performed the analyses, and drafted the manuscript. DAW and CNS revised the manuscript. All authors read and approved the final manuscript.

## Pre-publication history

The pre-publication history for this paper can be accessed here:


